# Measurement of Inositol 1,4,5-Trisphosphate in Living Cells Using an Improved Set of Resonance Energy Transfer-Based Biosensors

**DOI:** 10.1371/journal.pone.0125601

**Published:** 2015-05-01

**Authors:** Gergő Gulyás, József T. Tóth, Dániel J. Tóth, István Kurucz, László Hunyady, Tamas Balla, Péter Várnai

**Affiliations:** 1 Department of Physiology, Faculty of Medicine, Semmelweis University, Budapest 1094, Hungary; 2 Section on Molecular Signal Transduction, Program for Developmental Neuroscience, National Institute of Child Health and Human Development, National Institutes of Health, Bethesda, MD 20892, United States of America; Penn State Hershey College of Medicine, UNITED STATES

## Abstract

Improved versions of inositol-1,4,5-trisphosphate (Ins*P*_3_) sensors were created to follow intracellular Ins*P*_3_ changes in single living cells and in cell populations. Similar to previous Ins*P*_3_ sensors the new sensors are based on the ligand binding domain of the human type-I Ins*P*_3_ receptor (InsP_3_R-LBD), but contain a mutation of either R265K or R269K to lower their Ins*P*_3_ binding affinity. Tagging the Ins*P*_3_R-LBD with N-terminal Cerulean and C-terminal Venus allowed measurement of Ins*P*_3_ in single-cell FRET experiments. Replacing Cerulean with a Luciferase enzyme allowed experiments in multi-cell format by measuring the change in the BRET signal upon stimulation. These sensors faithfully followed the agonist-induced increase in Ins*P*_3_ concentration in HEK 293T cells expressing the Gq-coupled AT1 angiotensin receptor detecting a response to agonist concentration as low as 10 pmol/L. Compared to the wild type Ins*P*_3_ sensor, the mutant sensors showed an improved off-rate, enabling a more rapid and complete return of the signal to the resting value of Ins*P*_3_ after termination of M3 muscarinic receptor stimulation by atropine. For parallel measurements of intracellular Ins*P*_3_ and Ca^2+^ levels in BRET experiments, the Cameleon D3 Ca^2+^ sensor was modified by replacing its CFP with luciferase. In these experiments depletion of plasma membrane PtdIns(4,5)*P*_2_ resulted in the fall of Ins*P*_3_ level, followed by the decrease of the Ca^2+^-signal evoked by the stimulation of the AT1 receptor. In contrast, when type-III PI 4-kinases were inhibited with a high concentration of wortmannin or a more specific inhibitor, A1, the decrease of the Ca^2+^-signal preceded the fall of Ins*P*_3_ level indicating an Ins*P*_3_-, independent, direct regulation of capacitative Ca^2+^ influx by plasma membrane inositol lipids. Taken together, our results indicate that the improved Ins*P*_3_ sensor can be used to monitor both the increase and decrease of Ins*P*_3_ levels in live cells suitable for high-throughput BRET applications.

## Introduction

Inositol-1,4,5-trisphosphate (Ins*P*_3_) plays a central role in calcium signaling. Its production is catalyzed by phospholipase C enzymes activated through receptor stimulation. Measurements of Ins*P*_3_ kinetics for a long time relied upon isotope labeling of cell populations followed by extraction and HPLC-separation of the active (1,4,5) isomer from the inactive (1,3,4) one [1]. Mass measurements of Ins*P*_3_ using radio-receptor assays have also been used [2] to measure absolute mass changes, again from populations of cell, but these methods have been quite cumbersome and unable to provide very detailed time resolution. Moreover, these methods did not allow detection of kinetic changes in single cells with accurate comparisons with other parameters, such as cytoplasmic Ca^2+^ changes. These deficiencies prompted several groups, including ours, to develop Ins*P*_3_ sensor that would be useful for single-cell analysis. With the advent of fluorescent proteins and the development of fluorescence resonance energy transfer (FRET) technology, Ins*P*_3_ probes based on the ligand binding domain of the Ins*P*_3_ receptor have been introduced and used successfully in several single-cell applications [3–7].

In reviewing our experimental data obtained with a sensor that was developed in our group (using the same principles referenced above) and comparing it with published sensors, such as IRIS [3], we noted that the sensors not only distorted Ins*P*_3_ kinetics because of their buffering effects (a complication that is unavoidable with any probe), but they also showed Ins*P*_3_ kinetics suggestive of slow off-rates. To overcome this problem, we designed modified probes to address these kinetic deficiencies. Importantly, we also wanted to take this tool further such that it could be used in cell populations allowing a format amenable to screening applications.

Here we report on the fine-tuning and characterization of our Ins*P*_3_ sensor based on the human type-I Ins*P*_3_ receptor LBD (residues 224–605). Structural studies showed that Ins*P*_3_ binding leads to a conformational change of this protein domain, which can be translated to a change in FRET signal between two appropriate fluorophores placed at the two ends of the LBD. Similar probes have been introduced and published (see Table 1). It has been show earlier that deletion of the N-terminal 223 amino acids increases the affinity of the LBD, so the 224–605 LBD has a higher affinity than the native Ins*P*_3_ receptor channel [8]. Therefore, we decided to engineer slightly lower affinity mutants by mutating the Ins*P*_3_ binding site in order to improve its off-rate upon decrease in Ins*P*_3_ but still keep their abilities to detect the increase of Ins*P*_3_ level. In addition, we have demonstrated the ability of these probes to faithfully monitor Ins*P*_3_ concentrations by either FRET or bioluminescence resonance energy transfer applications (BRET).

**Table 1:** Ins*P*_3_ sensors in use for single-cell fluorescent energy transfer-based applications.

Sensor	Ins*P*_3_-binding domain	K_D_	Fluorescent proteins	Reference
**LIBRA**	rat type-III Ins*P*_3_R (1–604)	404 nM	CFP/YFP	[4, 31]
LIBRA-ΔN*	rat type-III Ins*P*_3_R (227–604)	ND	CFP/YFP	[4, 5, 31]
LIBRA-vI	rat type-I Ins*P*_3_R (1–604)	269 nM	CFP/Venus	[32]
LIBRA-vII	rat type-II Ins*P*_3_R (1–604)	234 nM	CFP/Venus	[32]
LIBRA-vIIS	LIBRAvII R440Q	117 nM	CFP/Venus	[32]
LIBRA-vIII	rat type-III Ins*P*_3_R (1–604)	492 nM	CFP/Venus	[5, 32]
LIBRA-vIIIS	LIBRAvIII R440Q	≈ 250 nM	CFP/Venus	[32]
				
**Fretino**	human type-I Ins*P*_3_R (224–579)	8 nM	CFP/YFP	[7]
Fretino-2	Fretino R504Q	190 nM	CFP/YFP	[7]
Fretino-3**	Fretino R508Q	ND	CFP/YFP	[7]
Fretino-4	human type-I Ins*P*_3_R (1–604)	ND	CFP/YFP	[7]
				
**IRIS-1**	mouse type-I Ins*P*_3_R (224–575)	549 nM (cell lysate), 437 nM (purified)	Venus/CFP	[3]
IRIS-1-Dmut**	IRIS-1 T276A, K508Q	ND		[3]
IRIS-1.2	IRIS-1 K249Q	3–4 μM		[3]
	mouse type-I Ins*P*_3_R (224–579)	95 nM	Venus/CFP	[3]
	mouse type-I Ins*P*_3_R (224–584)	105 nM	Venus/CFP	[3]
	mouse type-I Ins*P*_3_R (224–604)	107 nM	Venus/CFP	[3]
**FIRE-1**	rat type-I Ins*P*_3_R (1–589)	31 nM	CFP/YFP	[6]
FIRE-2	rat type-II Ins*P*_3_R (1–604)	ND	CFP/YFP	[6]
FIRE-3	rat type-III Ins*P*_3_R (1–604)	36 nM	CFP/YFP	[6]

K_D_ values represent the Ins*P*_3_ concentrations required to reach 50% of the dynamic range of the appropriate sensor. ND means not determined.

*Ins*P*_3_ insensitive mutant of LIBRA

**non-binding mutant for control experiments

To analyze and compare our sensors, we needed an experimental system in which an increase or decrease in Ins*P*_3_ concentration could be equally established. For this, type-1 angiotensin receptor (AT1R) or the M3 cholinergic receptor was transiently transfected into HEK 293T cells, so that Ins*P*_3_ concentration could be increased by angiotensin II or carbachol stimulation, respectively. Decrease of Ins*P*_3_ was evoked by terminating the muscarinic response by atropine. Under these conditions both the wild-type and the mutant sensors were able to show the rapid rise in Ins*P*_3_ levels, but the mutants were able to detect a more rapid and full decline in Ins*P*_3_ concentration. Our data suggest that these improved mutant sensors are suitable to investigate Ins*P*_3_ signaling more accurately than previous ones either by single-cell imaging or in cell population measurements.

## Materials and Methods

### Materials

Molecular biology reagents were obtained from Fermentas (Vilnius, Lithuania). Cell culture dishes and plates were purchased from Greiner (Kremsmunster, Austria). Coelenterazine *h* was purchased from Regis Technologies (Morton Grove, IL). Lipofectamine 2000 was from Invitrogen (Carlsbad, CA). Rapamycin was obtained from Merck (Darmstadt, Germany). GeneCellin transfection reagent was from BioCellChallenge (Toulon, France). Atropine was purchased from EGIS (Budapest, Hungary). Unless otherwise stated, all other chemicals and reagents were purchased from Sigma (St Louis, MO).

### DNA constructs

The R265K, R269K, R568K, R504K and R265,269K mutations were introduced by site-directed mutagenesis (Agilent Technologies, Santa Clara, CA USA) in the previously created mRFP-Ins*P*_3_R-LBD (residues 224–605 of human type-1 Ins*P*_3_ receptor S1+) construct used for bacterial expression of the fusion proteins [9]. To create the Ins*P*_3_ sensors first we made a FRET plasmid backbone by cloning the monomeric Venus [10] into the pEYFP-C1 plasmid, in which YFP was already replaced by Cerulean [11], using EcoRI and NotI enzymes. The wild type or mutant Ins*P*_3_R-LBDs were then inserted between the two fluorophores using XhoI and EcoRI resulting in two short linkers before and after the LBD (NEQRSR and NS). From these FRET sensors the BRET sensors were prepared by replacing Cerulean with super *Renilla* luciferase [12]. To improve the optical parameters, another set of BRET sensors were created by replacing Venus with the Venus cp173-Venus tandem used in other sensors like the Epac cAMP sensor [13].

The Ca^2+^ sensor used in the BRET measurements was created by replacing the Ins*P*_3_R-LBD with the appropriate sequence derived from Cameleon D3 [14]. For the replacement, first this sequence was amplified using PCR with the sense primer CTCGAGACCAACTGACAGAAGAGCAGATTGCAGAG and antisense primer GAATTCAGTGCCCCGGAGCTGGAGATCTTC, and then it was cloned into the BRET plasmid using XhoI and EcoRI enzymes.

Wild type human M3 cholinergic receptor (N-terminal 3x-hemagglutinin tagged) was purchased from S&T cDNA Resource Center (Rolla, MO). The non-internalizing rat type-I angiotensin receptor (AT1R-Δ319) was described earlier [15]. The plasma membrane targeted FRB-mRFP and mRFP-FKBP-5-ptase constructs used for rapamycin-induced PtdIns(4,5)*P*_2_ depletion were described earlier [16] with the difference that for plasma membrane targeting of the FRB protein, we used the N-terminal targeting sequence (1–10) of mouse Lck (GenBank accession number: NM_001162433) [17].

### Cell culture

HEK 293T and COS-7 cells (ATCC, Manassas, VA) were maintained in Dulbecco’s modified Eagle’s medium (DMEM, Lonza 12–604) supplemented with 10% fetal bovine serum, 50 U/ml penicillin and 50 μg/ml streptomycin in a 5% humidified CO_2_ incubator at 37°C in 10 cm tissue culture plastic dishes.

### Ins*P*_3_ binding experiments

mRFP-tagged wild type and mutant Ins*P*_3_R-LBD domains, built into the pET-23b bacterial expression vector (Novagen) were used to transform the BL-21 DE3 Star strain of *Escherichia coli* (Invitrogen). Bacterial cells were grown to A_600_ 0.6–0.9 at 37°C and induced with 300 μM isopropyl-1-thio-β-D-galactopyranoside at 18–20°C for 8 hours. Purification of the recombinant protein as well as Ins*P*_3_ binding assay were performed as described previously [9], using 0.75 μCi (1 nM) [^3^H]Ins(1,4,5)*P*_3_ (Amersham Biosciences) and 200 ng protein in an incubation volume of 50 μl.

### Fluorescence Resonance Energy Transfer (FRET) measurements

For FRET measurements HEK 293T cells were trypsinized and plated on poly-lysine-pretreated (0.001%, 1 hour) No 1.5 glass coverslips in 35 mm plastic dishes at 3x10^5^ cells/dish density. After one day the culture medium was changed to 1 ml Opti-MEM (Gibco) medium, and then 200 μl transfection solution containing the indicated DNA constructs (1 μg total DNA/dish) and 2 μl/dish Lipofectamine 2000 was added. After 6 hours 1 ml DMEM containing serum and antibiotics was added. Measurements were performed 24–32 hours after the transfection. Before the measurements the coverslips were placed into Attofluor cell chambers (Invitrogen) and the medium was changed to 800 μl of a modified Krebs–Ringer buffer containing 120 mM NaCl, 4.7 mM KCl, 1.2 mM CaCl_2_, 0.7 mM MgSO_4_, 10 mM glucose, and 10 mM Na-HEPES, pH 7.4. Measurements were performed at room temperature using an inverted microscope (Axio Observer D1, Zeiss, Germany) equipped with a 40x/1.3 oil-immersion objective (Plan-APO, Zeiss) and a Cascade II camera (Photometrics, Tucson, AZ). Excitation wavelengths (435 nm and 500 nm) were set by a monochromator connected to a 75 W Xenon lamp (DeltaRAM, Photon Technology International, Birmingham, NJ). The emitted light was separated by a dichroic beamsplitter (Chroma 69008bs), and then detected through the appropriate emission filters for Cerulean (470/24 nm) and Venus (535/30 nm). Images were acquired every 5 s. The indicated reagents were also dissolved in modified Krebs–Ringer buffer and were added manually in 200 μl, and mixed three times. The MetaFluor (Molecular Devices, Downingtown, PA) software was used for data acquisition, whereas for further data analysis including background subtraction, bleed through correction and 535/470 emission ratio calculation the MetaMorph (Molecular Devices) software was applied.

### Bioluminescence Resonance Energy Transfer (BRET) measurements

For BRET measurements HEK 293T cells were trypsinized and plated on poly-lysine-pretreated (0.001%, 1 hour) white 96-well plates at a density of 10^5^ cells/well together with the indicated DNA constructs (0.24–0.3 μg total DNA/well) and the cell transfection reagent (0.5 μl/well Lipofectamine 2000 or 1.5 μl/well GeneCellin). After 6 hours 100 μl/well DMEM containing serum and antibiotics was added. Measurements were performed 24–27 h after transfection. Before measurements the medium of cells was changed to a medium (50 μl) containing 120 mM NaCl, 4.7 mM KCl, 1.2 mM CaCl_2_, 0.7 mM MgSO_4_, 10 mM glucose, and 10 mM Na-HEPES, pH 7.4. Measurements were performed at 37°C using a Mithras LB 940 multilabel reader (Berthold, Germany). The measurements started with the addition of the cell permeable luciferase substrate, coelenterazine *h* (40 μl, final concentration of 5 μM), and counts were recorded using 485 and 530 nm emission filters. Detection time was 500 ms for each wavelength. The indicated reagents were also dissolved in modified Krebs–Ringer buffer and were added manually in 10 μl. For this, plates were unloaded, which resulted in an interruption in the recordings. All measurements were done in triplicates. BRET ratios were calculated by dividing the 530 nm and 485 nm intensities, and normalized to the baseline.

### Confocal microscopy

COS-7 cells were cultured on IBIDI μ-Slide 8 Well dishes (IBIDI GmbH, Cat. No.: 80826; 2x10^4^ cells/well) and transfected with the indicated constructs (1 μg DNA total/dish) using 0.5 μl/well Lipofectamine 2000 for 24 h. Confocal measurements were performed at 35°C in a modified Krebs-Ringer buffer described above, using a Zeiss LSM 710 scanning confocal microscope and a 63x/1.4 oil-immersion objective. Post-acquisition picture analysis was performed using the Photoshop (Adobe) software to expand to the full dynamic range but only linear changes were allowed.

### Statistical analysis

To calculate the half-time values (τ) of the decay phase, a curve fitting procedure was applied to the values in each individual experiment using the 3 parametric exponential decay equation of (y = y_0_+ae^-bx^). τ values were then averaged and subjected to a t-test.

## Results

### Generation of reduced affinity Ins*P*_3_ sensors that can be used in energy transfer measurements

**Fig 1:**
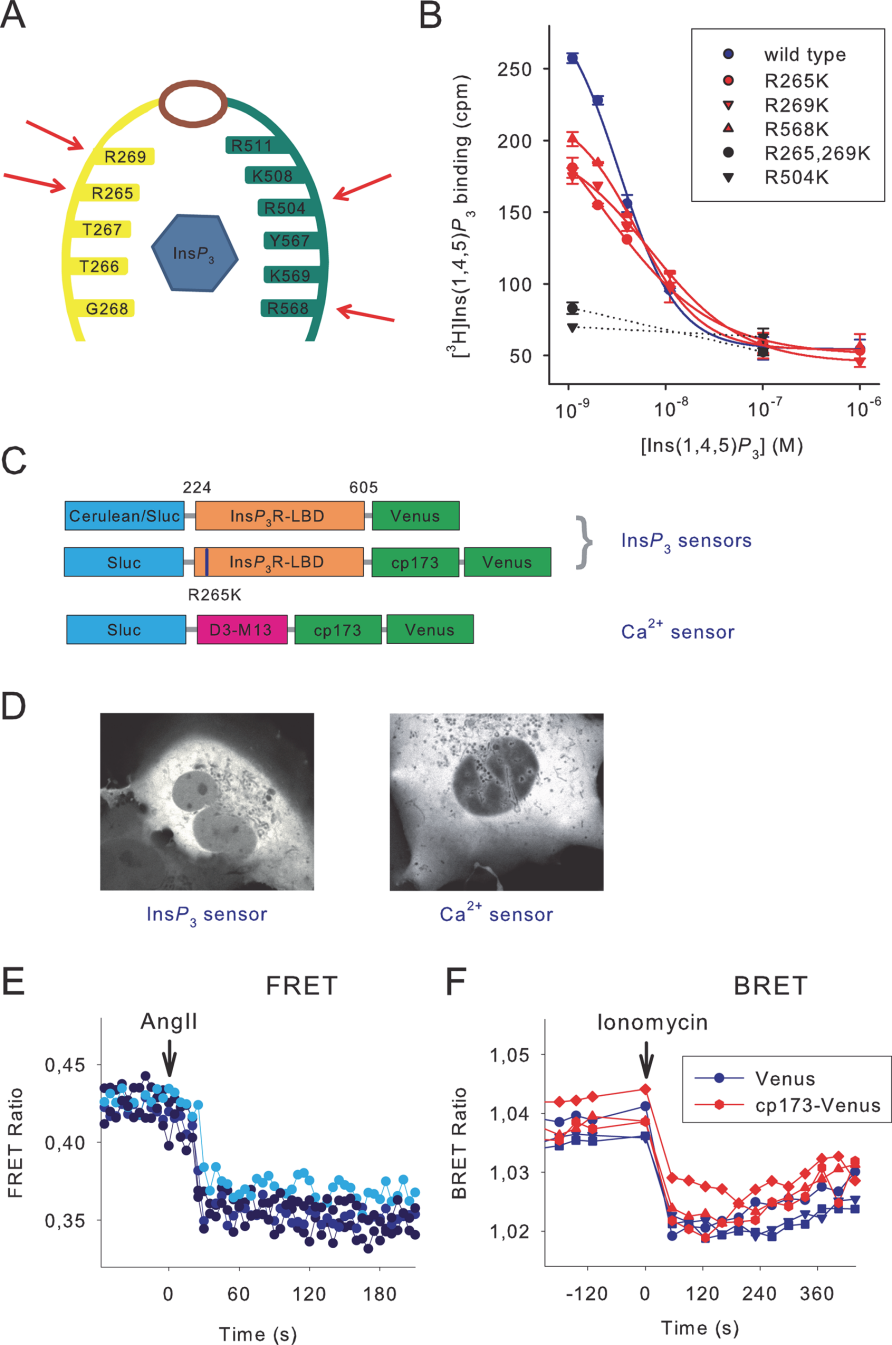
Characterization of the newly developed Ins*P*_3_ sensor. (A) Schematic drawing of the inositol 1,4,5-trisphosphate (Ins*P*_**3**_)-binding core domain based on its crystal structure [18]. Residues in the alpha helical domain and the beta-trefoil domain are highlighted in green and yellow, respectively. Ins*P*_**3**_ is highlighted in blue. The hinge region is shown in purple. Arginine to lysine mutations were introduced in the ligand binding residues at positions 265, 269, 504 and 568 (red arrows). (B) *In vitro* characterization of the inositol phosphate binding of recombinant mRFP-tagged Ins*P*_**3**_ binding domains. The assays were performed using [^3^H]Ins*P*_**3**_ (see methods) in the presence of the indicated concentrations of the respective unlabeled ligand. Two separate experiments in triplicates. (C) Schematic representations of domain structures of the different Ins*P*_**3**_ biosensors and a modified Cameleon D3 BRET sensor for measuring the changes in cytoplasmic Ca^2+^ concentration. The Ins*P*_**3**_ sensors contain Cerulean (FRET) or Sluc (BRET) on the N-terminus, the ligand-binding domain of the human type-I Ins*P*_**3**_-receptor and either Venus or circularly permuted Venus (cp173) and Venus on the C-terminus. The blue lines show the approximate location of the designed mutations (R265K). The Ca^2+^ sensor contains the MLCK calmodulin binding peptide M13 and the D3 variant of calmodulin. (D) Representative images of Ins*P*_**3**_ or Ca^2+^ biosensor-containing COS-7 cells. (E) Measurements of FRET in individual HEK 293T cells expressing the FRET Ins*P*_**3**_ biosensor and the AT1 angiotensin receptor. Ins*P*_**3**_ production of the cells was triggered by 1 μM angiotensin II (Ang II). Note that binding of Ins*P*_**3**_ resulted in a decrease of the energy transfer. (F) Measurement of BRET in HEK 293T cells expressing two types of BRET Ins*P*_**3**_ biosensors containing a single or a tandem fluorescent protein (Venus or cp173-Venus and Venus). Ins*P*_**3**_ production was induced by ionomycin (10 μM). The curves indicate the raw BRET ratios of individual wells of a 96-well white tissue culture plate used in the BRET measurements.

To design an improved Ins*P*_3_ sensor first we decided to perform a moderate modification in the Ins*P*_3_ binding domain (224–605 amino acids) of human type-I Ins*P*_3_ receptor (IP3R-LBD) [18] by replacing specific arginine residues to lysines. Since the side chain of lysine is shorter than that of arginine and lysine is a less basic amino-acid than arginine (pI-values are 9.74 and 10.76, respectively) we reasoned that interaction of the mutant protein with negatively charged phosphate groups of Ins*P*_3_ will be weaker. We expected that this manipulation would decrease the binding affinity and therefore increase the off-rate performance of the sensor. Based on the crystal structure of the binding domain [19] we selected R265, R269, R504 and R568 residues for mutation (Fig 1A). To investigate the Ins*P*_3_ binding properties of these proteins, first we created constructs for bacterial expression. The mRFP-, and 6xHis-tagged Ins*P*_3_ binding domains were purified on Ni^2+^ columns, and in vitro binding assays were performed using [^3^H]Ins*P*_3_ as the tracer. As shown in Fig 1B the binding of R265K, R269K and R568K were weaker compared to the wild type protein, while the R504K and the double mutant R265,269K were very poor Ins*P*_3_ binders. Scatchard analysis of the curves also showed the lower affinity of the mutants: the K_D_ value for the R265K, R269K and R568K mutants were 6.06 nM, 10.07 nM and 5.30 nM, respectively compared to 3.04 nM for the wild type LBD. Based on these results, the R265K and R269K mutants were selected for further analysis.

In previous studies we created the Ins*P*_3_R-LBD with various N-terminal fluorescent tags, such as mRFP or Cerulean, in combination with a C-terminal Venus tag. Application of the protein as Ins*P*_3_ sensor in BRET measurements requires that the N-terminal fluorescent protein tag be replaced with a luciferase enzyme. Fig 1C shows the final structure of the Ins*P*_3_ sensors that contain either Cerulean [11] or Super Luciferase (Sluc) [12] tag on their N-terminus, and Venus on the C-terminus. Studies on the dependence of energy transfer efficiency between fluorescent proteins suggested that introduction of double-acceptor moieties may significantly enhance the FRET efficiency value [13]. Therefore, we also created constructs with tandem yellow fluorescent tag (cp173 Venus and Venus) on their C-termini [13].

As shown before for the previously developed sensors, expression of these sensors in mammalian cells (COS-7 and HEK 293T) resulted in uniform cytoplasmic distribution of the fusion proteins and their reduced levels in the nucleus (Fig 1D). Similarly to other published Ins*P*_3_ sensors (see Table 1), Ins*P*_3_ binding caused a decrease of energy transfer in both FRET and BRET measurements (Fig 1E and 1F). Notably, duplication of the acceptor did not result in improvement of the signal in the BRET format (Fig 1F). Since raw BRET ratio values often show a spontaneous increase over time, we included wells that were left unstimulated and used these to correct for baseline drift. From these data the reciprocal of the I/I_0_ values were calculated and plotted, therefore the elevation of cytoplasmic Ins*P*_3_ level corresponds to an increased value on the graphs. Since the BRET application is done in cell suspension and therefore inherently reports on averaged cell responses, the subsequent characterization was done using the BRET approach.

### Characterization of the Ins*P*_3_ sensitivity and reversibility of the reduced affinity Ins*P*_3_ sensors

**Fig 2:**
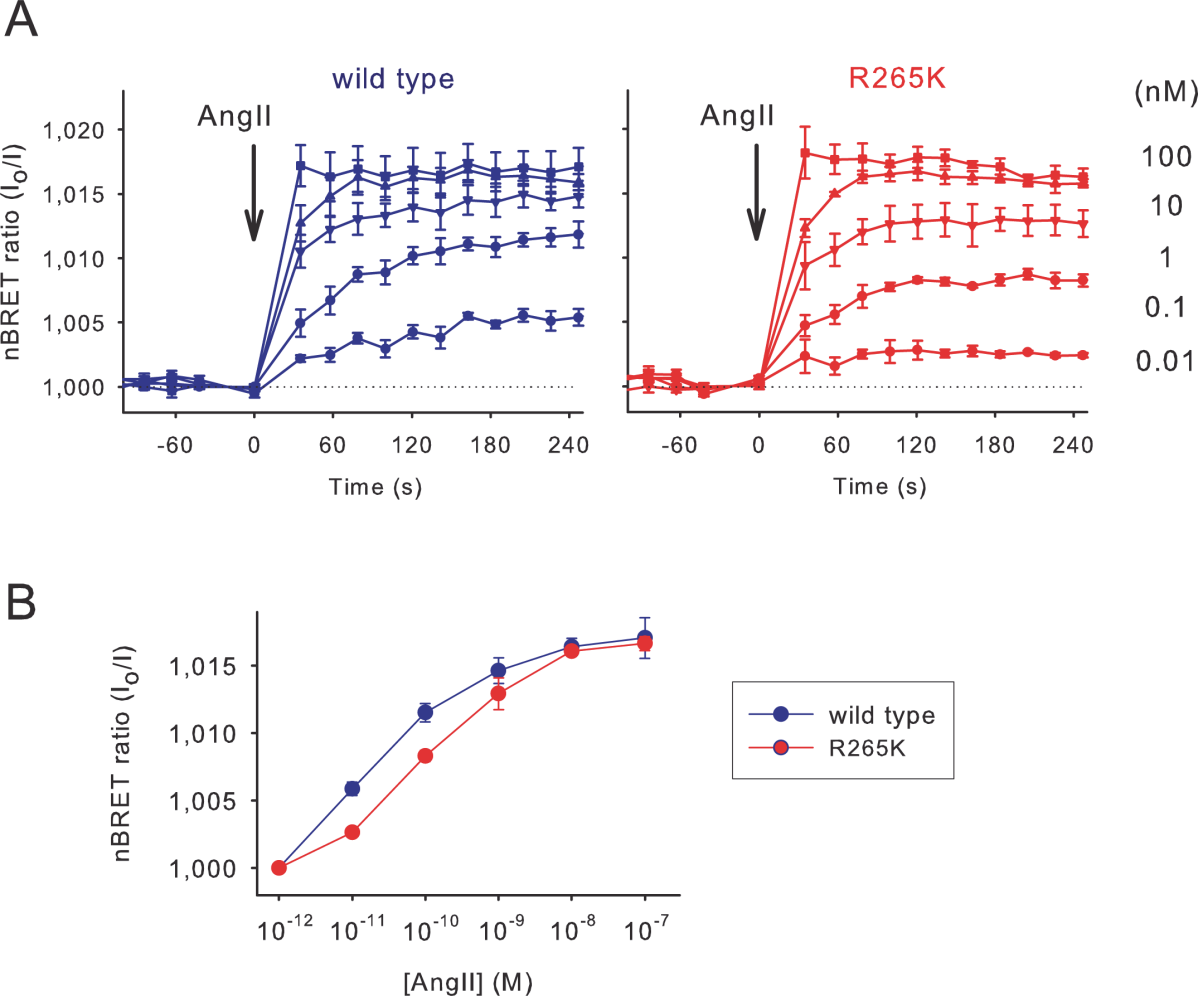
Comparison of the activation properties of the wild type and low-affinity Ins*P*_3_ sensors. (A) Normalized BRET ratio (reciprocal of I/I_**0**_) measured in HEK 293T cells transiently transfected with the AT1 angiotensin receptor and the wild type or R265K mutant Ins*P*_**3**_ biosensors upon Ang II stimulation (10^-12^-10^-7^ M) added manually. To avoid desensitization of the receptor a non-internalizing receptor was used (AT1R-Δ319). Error bars show standard error values from three independent measurements performed in triplicate. (B) Concentration-response curves for Ang II. Ins*P*_**3**_ responses were measured 5 minutes after stimulation. The right shift caused by the R265K mutation corresponds to the lower ligand binding affinity of the Ins*P*_**3**_ biosensor.

To compare the Ins*P*_3_-induced signals of the wild type and low affinity Ins*P*_3_ sensors, HEK 293T cells were transfected with the cDNAs of both the human AT1 receptor and the Sluc/Venus version of the appropriate Ins*P*_3_ sensor. BRET changes were then recorded following angiotensin II (Ang II) stimulation. As shown on Fig 2A, increasing concentrations of Ang II from 10^–12^ to 10^–7^ M resulted in an increasing BRET signal in case of both the wild type and the mutant sensors. The signals also showed a kinetic difference as the concentration of Ang II was increased (Fig 2A). While the maximal response was the same, a moderate shift to the right occurred on the dose-response curve in case of the sensor with the R265K mutation reflecting the lower affinity of this mutant sensor (Fig 2B).

**Fig 3:**
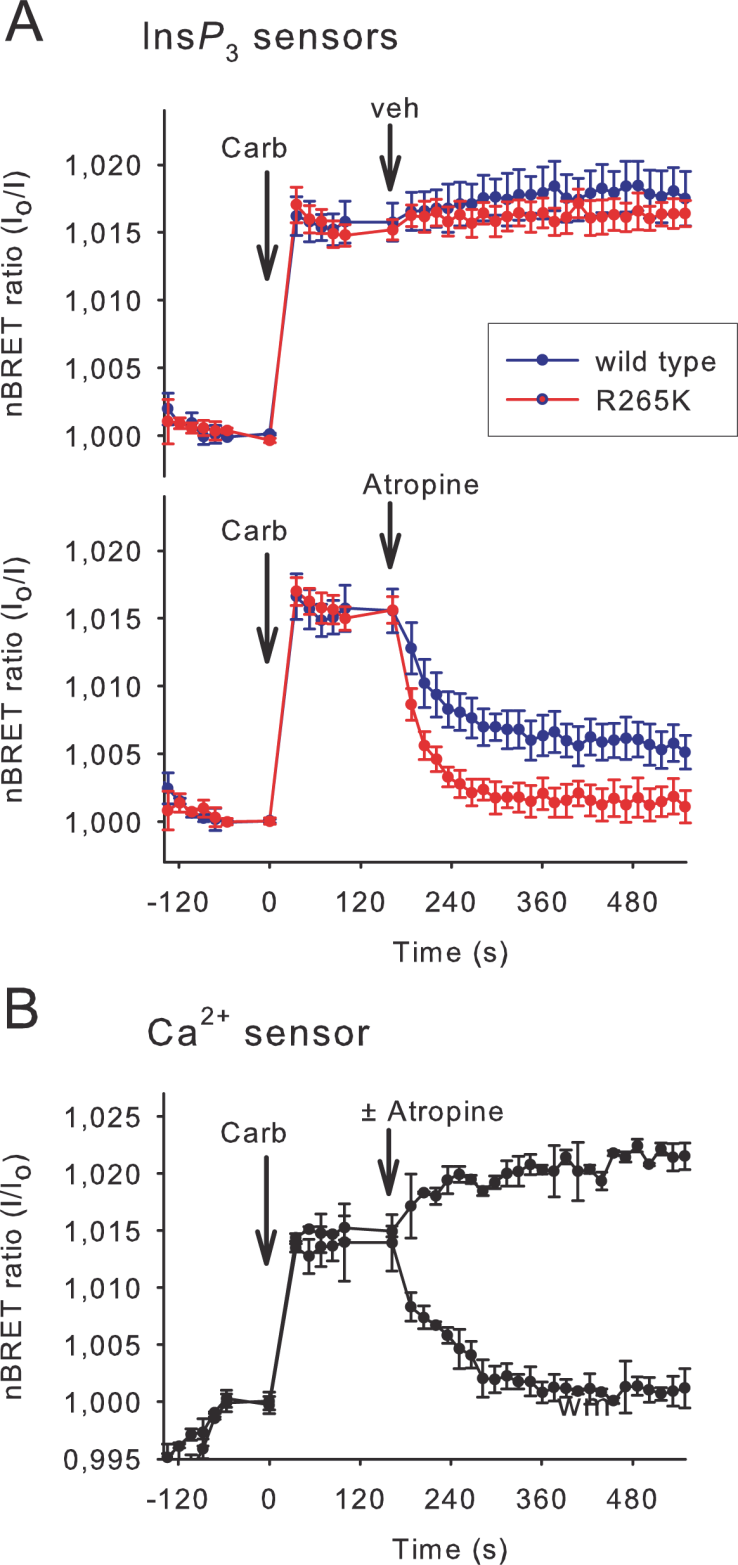
Comparison of the reversibility of the wild type and low-affinity Ins*P*_3_ sensors. (A) Ins*P*_**3**_ production was evoked in HEK 293T cells expressing the M3 cholinergic receptor and various BRET Ins*P*_**3**_ biosensors. Stimulation of the cells with 10 μM carbachol (Carb) added manually resulted in a maximal Ins*P*_**3**_ response sustained for at least 10 minutes (upper graph) as indicated by both the wild type and the R265K mutant Ins*P*_**3**_ biosensors. To examine how the biosensors can monitor the decrease of the Ins*P*_**3**_ signal the activation process was stopped by 10 μM atropine (lower graph) added manually, which caused the divergence of the curves corresponding to the wild type and R265K mutant Ins*P*_**3**_ biosensors. (B) Parallel measurement of the cytoplasmic Ca^2+^ level measured with the modified Cameleon D3 BRET sensor. Experiments were carried out on the same plate in parallel wells containing cells expressing the Ca^2+^ sensor instead of the Ins*P*_**3**_ sensors. Values are means ± SE of three independent experiments performed in triplicate.

To investigate the reversibility of the Ins*P*_3_ binding of the sensors, HEK 293T cells transiently expressing the M3 cholinergic receptor were stimulated first with carbachol (10 μM), which activated the Gq signaling pathway and elevated the cytoplasmic Ins*P*_3_ and Ca^2+^ levels. These responses can be quickly terminated by adding the competitive antagonist atropine (10 μM). As shown in Fig 3A the rising phases of the signals, which reflect the elevation of Ins*P*_3_, were indistinguishable between the wild-type and mutant Ins*P*_3_ sensors (however, note the low temporal resolution of about 3 points per minute). In contrast, the mutant sensor showed a significantly enhanced off-rate (τ = 23.0±2.3 s) compared to the wild type sensor (τ = 44.9±7.5 s) (Mean ± S.E.M. n = 5, p = 0.024), and a complete return to the baseline. Ins*P*_3_ measurement performed with the wild-type sensor revealed an elevated level of cytoplasmic Ins*P*_3_ concentration after the atropine treatment, which may reflect an incomplete dissociation of Ins*P*_3_ from the high affinity wild-type sensor or a non-complete inhibition of M3R signaling with atropine. Notably, in control cells, which were not treated with atropine, the Ins*P*_3_ level remains elevated up to 10 minutes consistent with a slow desensitization of the M3R, as demonstrated previously [20].

Parallel measurements of cytoplasmic Ca^2+^ concentration, performed under the same experimental condition (in the same plate, but in cells that expressed the BRET version of a previously described low K_D_ Ca^2+^ sensor [14] instead of the Ins*P*_3_ sensors), revealed the termination of the Ca^2+^ signal upon atropine treatment with a kinetic, which was highly similar to the one showed by the mutant Ins*P*_3_ sensor (Fig 3B).

### Parallel cytoplasmic Ins*P*_3_ and Ca^2+^ measurements suggest a role of phosphoinositides in the generation of capacitative Ca^2+^ entry

Having performed these control experiments we wanted to apply these new sensors to address some lingering questions in Ins*P*_3_/Ca^2+^ signaling. It has been widely accepted that a sustained elevation in cytoplasmic Ca^2+^ upon stimulation with a Ca^2+^-mobilizing agonist is the consequence of emptying the intracellular Ca^2+^ stores, with a sequential activation of Ca^2+^ influx into the cells via the Ca^2+^ channel Orai1 [21]. However, the role of phosphoinositides has been raised not only as the source of Ins*P*_3_ but also as possible regulators of the capacitative Ca^2+^ entry process [22].

**Fig 4:**
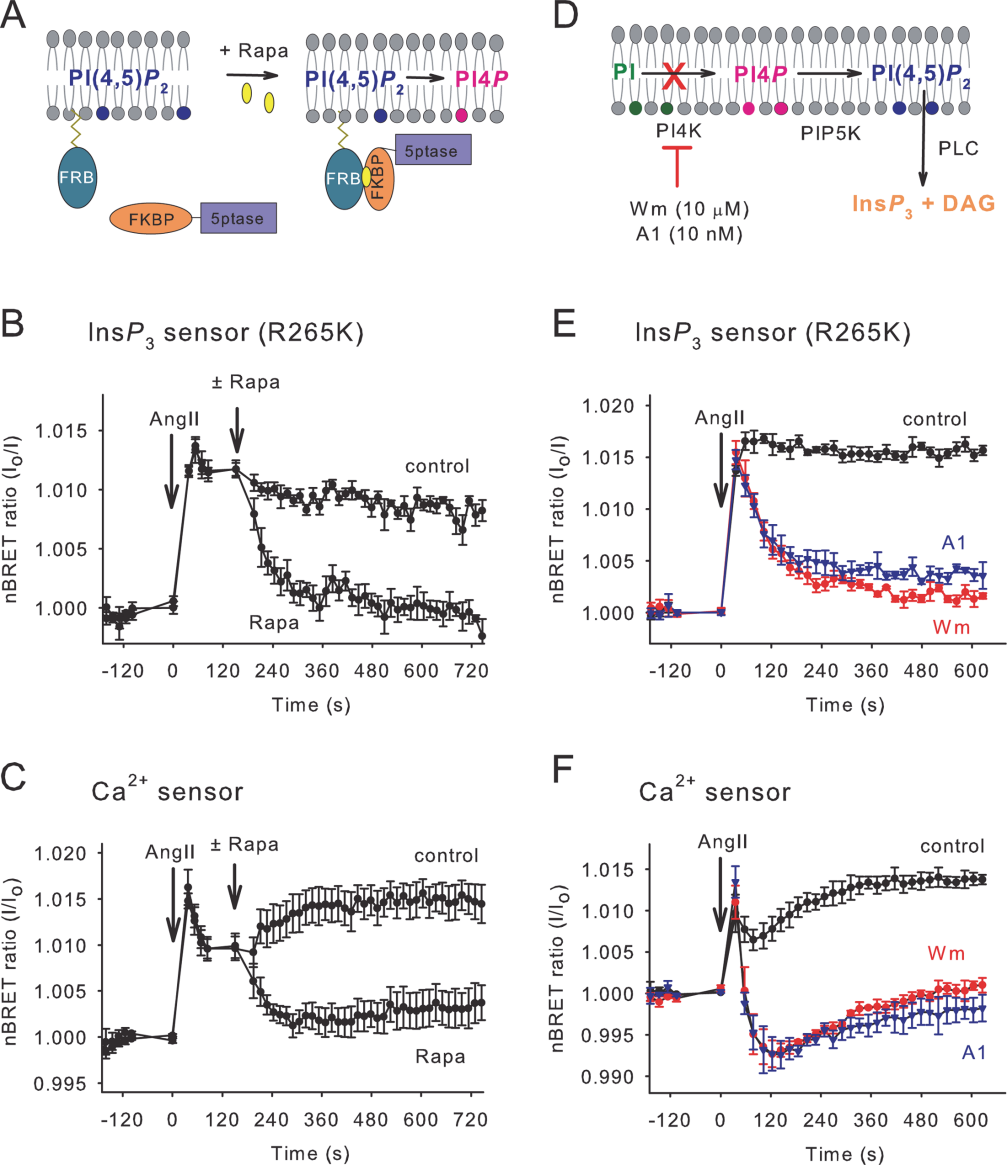
Reduction of plasma membrane PtdIns(4)*P* and PtdIns(4,5)*P*_2_ levels and their effect on the Ins*P*_3_ and Ca^2+^ signals upon hormonal stimulation. (A) Schematic representation of the plasma membrane PtdIns(4,5)*P*_**2**_ depletion system [33]. Addition of rapamycin induces the heterodimerization of the FRB and FKBP domains in PM-FRB and FKBP-5ptase and thus causes the translocation of the latter molecule to the plasma membrane where it degrades PtdIns(4,5)*P*_**2**_. (B and C) HEK 293T cells were transiently transfected with AT1R-Δ319 together with the PtdIns(4,5)*P*_**2**_ depleting system and the indicated BRET sensors. After hormonal stimulation (100 nM Ang II), 300 nM rapamycin was added manually to cause acute depletion of PtdIns(4,5)*P*_**2**_ in the PM. (means ± SE, n = 4). (D) During the synthesis of PtdIns(4,5)*P*_**2**_, first the PI4K enzymes phosphorylate PI, and then PIP5K produces PtdIns(4,5)*P*_**2**_ from PtdIns(4)*P*. The plasma membrane PtdIns(4)*P* content can be depleted by preincubation of the cells with the selective PI4KIIIα inhibitor, A1 or a high concentration of wortmannin (Wm). (E and F) HEK 293T cells were transiently transfected with AT1R-Δ319 together with the indicated BRET sensors. A 10-minute preincubation with 10 μM wortmannin or 10 nM A1 was used to decrease the PtdIns(4)*P* level of the PM. The curves show the changes in the normalized BRET ratio (means ± SE, n = 3) of the indicated sensors upon manual addition of 100 nM Ang II.

To investigate the role of inositol phospholipids and Ins*P*_3_ in this process, we performed parallel measurements of cytoplasmic Ins*P*_3_ and Ca^2+^ levels under conditions, where either the plasma membrane (PM) phosphatidylinositol 4,5-bisphosphate [PtdIns(4,5)*P*_2_] level was rapidly reduced by the rapamycin-inducible PM PtdIns(4,5)*P*_2_-depletion system (Fig 4A) or the PtdIns(4)*P* supply was decreased by wortmannin pretreatment (Fig 4D). To avoid the complicating effects of receptor desensitization, HEK 293T cells expressing the rapamycin-regulated phosphatase system were transfected with the cDNA of a C-terminally tail-deleted non-internalizing AT1 receptor mutant (AT1R-Δ319) [15]. As shown on Fig 4B, stimulation with Ang II resulted in a large and sustained increase in cytoplasmic Ins*P*_3_ levels, which was rapidly terminated upon PtdIns(4,5)*P*_2_ depletion evoked by rapamycin addition. The decay of the Ins*P*_3_ signal correlated well with the decline of the cytoplasmic Ca^2+^ level (Fig 4C) measured in parallel (side by side on the same plate) using the BRET version of the original Cameleon D3 Ca^2+^ sensor [14]. This correlation indicates that the newly developed Ins*P*_3_ sensor is suitable for monitoring the rapid termination of this signaling event.

Next we compared Ins*P*_3_ and Ca^2+^ kinetics following Ang II stimulation of cells pretreated with either 10 μM wortmannin or 10 nM A1, a recently published potent and more specific PI4KIIIα inhibitor [23]. Wortmannin at this concentration inhibits type-III PI4Ks (both α and β), thereby limiting the replenishment of the phosphoinositide pools [24]. As shown on Fig 4E, both the Ang II-evoked Ins*P*_3_ and Ca^2+^ responses became transient when cells were pretreated with 10 nM A1 or 10 μM wortmannin for 10 minutes before Ang II stimulation, whereas the initial increases were very similar. This is in agreement with the earlier observation using isotope-based Ins*P*_3_ measurements, in which wortmannin pretreatment greatly reduced resting PtdIns(4)*P* but only slightly affected the prestimulatory PtdIns(4,5)*P*_2_ level [24]. However, strikingly, the decay in Ins*P*_3_ level was much slower than the decline in the Ca^2+^ levels after the pretreatment with PI4K inhibitors (Fig 4F). This suggested that the rapidly declining Ca^2+^ cannot be simply caused by the depletion of PtdIns(4,5)*P*_2_ as a cause of declining Ins*P*_3_ and refilling of the endoplasmic reticulum (ER) Ca^2+^ stores. These results suggested that PI4K inhibition had an additional effect on Ca^2+^ signals, most likely on entry via the store-operated pathways.

## Discussion

The purpose of the present studies was to develop an Ins*P*_3_ sensor that allows kinetic measurement of cytoplasmic Ins*P*_3_ concentration both in singe cells and in cell populations. The conformational change evoked by Ins*P*_3_ binding to the LBD domain of the Ins*P*_3_ receptor served as a rational basis for the development of various energy transfer-based Ins*P*_3_ sensors, summarized in Table 1. The evolution and evaluation of these sensors clearly showed that their Ins*P*_3_ binding affinity is a crucial factor in their suitability. While using the high affinity wild-type LBD domain in these applications makes the sensors very sensitive, their buffering effects and their slow off-rate has limited their use, especially in experiments where assessment of Ins*P*_3_ decreases was important. Several approaches have been used to modify the binding affinity of the LBD. For example, keeping the N-terminal inhibitory domain or truncation of the C-terminus of the LBD both reduced the binding affinity (Table 1). Mutations of amino acids responsible for the interaction with the Ins*P*_3_ ligand can either increase or decrease the affinity of ligand interaction. Unfortunately, the impact of such modifications often is too drastic and results in unsuitable sensors. In our studies the starting point was the binding domain (amino acids 224–605) of the human type-I Ins*P*_3_ receptor, which we previously used as a cytoplasmic Ins*P*_3_ buffer [25]. *In vitro* binding measurements performed with tritium-labelled Ins*P*_3_ resulted in an IC_50_ value of 4 nM for the ligand binding affinity of the GFP-tagged version of this domain [9], which is in good agreement with the 3 nM, calculated here from the Scatchard analysis for the ligand binding of the mRFP-tagged LBD. Since adding the N-terminal inhibitory domain led to a large drop in binding affinity (about one order of magnitude) [25], we attempted to make a more subtle change in the LBD, replacing key arginines within the Ins*P*_3_ binding site with lysines, trying to mimic the affinity close to that of the endogenous receptor. Among several mutants tested, the R265K and R269K mutants has proven to be suitable candidates for the development of improved Ins*P*_3_ sensors.

For FRET applications the wild type, R265K and R269K mutant LBDs were tagged with monomeric versions of Cerulean and Venus on their N- and C-termini, respectively. To make the sensor suitable for use in BRET measurements, we replaced Cerulean with Luciferase. The advantage of BRET measurement over FRET is that it can be carried out in simple plate readers and that it shows the average change in a cell population. Since it does not require excitation, there is no crosstalk between the emission channels. Therefore, energy transfer can be followed by simply calculating the emission ratio without any correction. Data processing is reduced only to taking the reciprocal of the ratio values to obtain an increase when Ins*P*_3_ level elevates, and normalizing the data to the resting values. Replacement of Venus with a double Venus construct (Venus cp173-Venus) did not yield further benefits either in the FRET or BRET format. Our mutant Ins*P*_3_ sensors tested in BRET applications showed a slightly reduced but still high sensitivity to agonist stimulation, while they performed better when the decay of Ins*P*_3_ was followed after termination of Ins*P*_3_ production. This was either achieved by terminating receptor stimulation or rapidly removing PtdIns(4,5)*P*_2_, the precursor of Ins*P*_3_. This allowed us to perform specific experiments to test the utility of these sensors to address questions regarding the connection between Ins*P*_3_ and Ca^2+^ entry regulation.

It has been shown previously that pretreatment of cells with concentrations of wortmannin, that inhibit PI3- and PI4-kinases, modifies the shape of the agonist-evoked Ca^2+^ signal by eliminating its sustained elevation [24, 26]. This was initially attributed to the fact that wortmannin limits the replenishment of the plasma membrane phosphoinositide pools by inhibiting type-III PI4Ks, therefore leading to the run-down of these lipids during agonist stimulation [24]. Since Ca^2+^ influx through the capacitative Ca^2+^ entry pathway is regulated by the ER luminal Ca^2+^ concentration, which in turn, is controlled by the opening of the Ins*P*_3_ receptor channels in the ER, it was logical to assume that the falling Ins*P*_3_ levels in wortmannin-treated cells would allow ER Ca^2+^ pools to refill and shut down the store-operated Ca^2+^ entry process. In fact, it has been shown by early studies that Icrac (the current that corresponds to store-regulated Ca^2+^ entry) is inhibited by wortmannin [27]. Subsequent studies performed after the identification of the molecules responsible for SOCE, have also indicated that phosphoinositides in the plasma membrane may directly influence this Ca^2+^ entry route either by affecting the Orai channels or the ER-sensor STIM1 molecule [22, 28–30].

Our new ability of monitoring Ins*P*_3_ and Ca^2+^ changes allowed us to address this question in a way that relied upon endogenous levels of STIM1 and Orai1.

We compared the decay kinetics of Ca^2+^ and Ins*P*_3_ either after rapidly removing PtdIns(4,5)*P*_2_ or limiting phosphoinositide supplies by wortmannin (or now the more specific PI4KIIIa inhibitor, A1) pretreatment. We found a significant difference between the two manipulations: while the Ins*P*_3_ and Ca^2+^ decreases were parallel after PtdIns(4,5)*P*_2_ depletion, the Ca^2+^ decrease was substantially faster than that of Ins*P*_3_ in the PI4K inhibitor pretreated cells. This suggested that PI4KIIIa inhibition exerted an additional effect on Ca^2+^ signaling. that was different from a simple PtdIns(4,5)*P*_2_ depletion evoked by the PM-recruited 5-ptase.

The most important difference between the two manipulations is the change in the level of plasma membrane PtdIns(4)*P*. While PtdIns(4,5)*P*_2_ elimination by the 5-ptase increases PtdIns(4)*P* levels, A1 or wortmannin pretreatment selectively depletes PtdIns(4)*P* even before agonist stimulation and PtdIns(4,5)*P*_2_ decreases only occur after agonist addition. All of these argue for a role for PtdIns(4)*P* or a mechanism sensitive to its level in playing a role in the control of store-operated Ca^2+^ entry. These conclusions were consistent with those of earlier reports using different approaches [22, 27].

We also observed a peculiar decrease of the Ca^2+^ levels below the prestimulatory values when cells were stimulated after PI4K inhibition. While this may be a feature brought out by our genetically encoded Ca^2+^ sensor, it raised the possibility that Ca^2+^ extrusion is also stimulated by agonists and together with inhibition of Ca^2+^ entry due to the simultaneous elimination of both PtdIns(4)*P* and PtdIns(4,5)*P*_2_ manifests in a Ca^2+^ decrease below basal. Further exploration of this possibility, however, was beyond the scope of this study.

In summary, in this paper we described and characterized an improved Ins*P*_3_ sensor which is based on the ligand binding domain of human type-1 Ins*P*_3_ receptor but contains the R265K mutation. While this sensor maintains its sensitivity and can be used to monitor small increases of cytoplasmic Ins*P*_3_ concentration, it also has an improved off-rate to follow the decrease of the Ins*P*_3_ level. While the sensor also works in FRET applications, an important new feature was its adaptation for BRET applications usable in plate readers to support high-throughput measurement of cytoplasmic Ins*P*_3_ in live cells alone, or in combination with measurements of other signaling factors, such as cytoplasmic Ca^2+^ concentration.
